# Global Distribution of *Babesia* Species in Questing Ticks: A Systematic Review and Meta-Analysis Based on Published Literature

**DOI:** 10.3390/pathogens10020230

**Published:** 2021-02-19

**Authors:** ThankGod E. Onyiche, Cristian Răileanu, Susanne Fischer, Cornelia Silaghi

**Affiliations:** 1 Department of Veterinary Parasitology and Entomology, University of Maiduguri, P. M. B. 1069, Maiduguri 600230, Nigeria; et.onyiche@unimaid.edu.ng; 2 Institute of Infectology, Friedrich-Loeffler-Institut, Federal Research Institute for Animal Health, Südufer 10, 17493 Greifswald-Insel Riems, Germany; cristian.raileanu@fli.de (C.R.); susanne.fischer@fli.de (S.F.); 3 Department of Biology, University of Greifswald, Domstrasse 11, 17489 Greifswald, Germany

**Keywords:** *Babesia*, questing tick, global, prevalence, molecular, meta-analysis

## Abstract

Babesiosis caused by the *Babesia* species is a parasitic tick-borne disease. It threatens many mammalian species and is transmitted through infected ixodid ticks. To date, the global occurrence and distribution are poorly understood in questing ticks. Therefore, we performed a meta-analysis to estimate the distribution of the pathogen. A deep search for four electronic databases of the published literature investigating the prevalence of *Babesia* spp. in questing ticks was undertaken and obtained data analyzed. Our results indicate that in 104 eligible studies dating from 1985 to 2020, altogether 137,364 ticks were screened with 3069 positives with an estimated global pooled prevalence estimates (PPE) of 2.10%. In total, 19 different *Babesia* species of both human and veterinary importance were detected in 23 tick species, with *Babesia microti* and *Ixodes* *ricinus* being the most widely reported *Babesia* and tick species, respectively. Regardless of species, adult ticks with 2.60% had the highest infection rates, while larvae had the least with 0.60%. Similarly, female ticks with 4.90% were infected compared to males with 3.80%. Nested-polymerase chain reaction (PCR) 2.80% had the highest prevalence among the molecular techniques employed. In conclusion, results obtained indicate that *Babesia* species are present in diverse questing tick species at a low prevalence, of which some are competent vectors.

## 1. Introduction

Both *Theileria* and *Babesia* species belong to the order Piroplasmida, are widely distributed and are among the economically important tick-borne hemoparasites of mammals [1]. Babesiosis has been well-known since the 19th century and is distributed worldwide as a disease of veterinary importance in cattle, sheep, pigs, dogs, and horses and in recent times has attracted attention as a zoonotic infection in humans [2,3].

*Babesia* is second only after *Trypanosomes* globally as the commonly found hemoparasites in the blood of mammals [4]. In 1888, Victor Babes, a Romanian biologist, was the first to discover the presence of intra-erythrocytic microorganisms in the blood of cattle, and he later observed similar intra-erythrocytic organisms in the blood of sheep [5]. A few years later, these microorganisms, which were later named “*Babesia*”, were noted in the blood of cattle in the United States [6]. These microorganisms in cattle were named *Babesia bovis* and *B. bigemina,* and in sheep, *B. ovis* [7]. Ever since, different species of *Babesia* have been observed parasitizing the blood of domestic animals. Over 100 species have been described thanks to the advances in microscopy, cell culture, and molecular techniques [1,3]. The clinical manifestations of babesiosis vary considerably across different animal species, but abortions, decreased milk and meat production, and mortality have been observed [8].

Furthermore, human babesiosis was first documented in the former Yugoslavia republic in 1957 [9]. Babesiosis in humans is becoming a public health concern as several species, including *B. microti*, *B. divergens* and *B. venatorum*, can infect humans accidentally, causing disease [8]. *Babesia microti* infections are less acute compared with *B. divergens*, while those due to *B. venatorum* are milder [10]. Affected persons are often asymptomatic except in immunocompromised individuals where the outcome can be fatal [8,11]. Clinical complications, such as hemolysis, acute respiratory distress and multiorgan malfunctioning leading to death have been observed [12].

Ixodid ticks are obligate hematophagous acarines, which feed on a wide variety of hosts, and over 700 species have been described [13]. To complete their life cycle, ticks must look for suitable hosts. Therefore, newly hatched larvae, nymphs and adults that are unfed need to seek a host for a blood meal for their further development into the next stage [14]. Detection and attachment to potential hosts in Ixodidae can be achieved through three major behavioral patterns: hunting, tick-host cohabitation, and questing [15].

Species of questing ticks within the genera *Ixodes*, *Dermacentor* and *Haemaphysalis* have been described and collected for the detection of tick-borne pathogens. Other species within the genus *Rhipicephalus* and *Hyalomma* have also been collected from the environment [16]. Questing ticks can be collected principally by flagging or dragging, among other methods, including trapping using baits (e.g., carbon dioxide) [14]. Ixodid ticks are the primary vectors of *Babesia*, but the parasites are sustained in a complex system of animal reservoirs and tick vectors [17,18]. In Ixodid ticks, the sexual phase of the life cycle of *Babesia* typically takes place acquiring and transmitting the parasites during blood meals from their host [19,20]. Transovarial transmission is exclusive within the *Babesia* sensu stricto evolutionary lineage, thereby allowing the pathogens to perpetuate their long-term persistence in ticks and serving as parasite reservoirs when vertebrate hosts are absent [18,20].

*Ixodes ricinus* is the most common tick, widely distributed in Europe (Western Palearctic), while the focal distribution of *Dermacentor reticulatus* has been observed [17,21,22,23,24,25]. Other species like *I. scapularis* are common in the United States of America [26,27], *I. ovatus* and *Hemaphysalis longicornis* in East Asia [28,29,30] and *I. persulcatus* in Europe (Russia) and parts of Central and Northern Asia [31,32]. Other species of Rhipicephalid ticks have also been reported globally [8,33,34].

Major interest in the role of questing ticks as vectors of pathogens of zoonotic importance began to emerge in the early 2000s. In questing ticks, aside from *B. microti,* which has been well reported in Europe, Asia, and America with varying infection rates [35,36,37,38], *B. divergens* and *B. venatorum* have been exclusively reported in Europe in the last two decades [17,39,40]. Other species of *Babesia* that infect domestic animals and that have been detected in questing ticks include *B. canis* [24,41], *B. odocoilei* [26,42], *B. ovata* [29,43], *B. bigemina* [8,43], *B. bovis* [43,44], *B. caballi* [41,45], *B. capreoli* [17,46,47] and many more.

In the last two decades, several individual studies around the world attempted to screen for the presence of *Babesia* species in questing ticks using molecular techniques, but no attempt has been made to synchronize the results from all these studies. Assessing the global state of the pathogen prevalence in unfed host-seeking ticks is essential to develop effective control measures. Therefore, in this study, we undertook a comprehensive assessment to determine the occurrence of *Babesia* species in unfed host-seeking ticks collected from vegetation while using globally published epidemiological data. To achieve the above aim, we evaluated prevalence rates according to tick species, region of sampling, life stages of ticks, sex of adult ticks, sampling years and molecular detection techniques.

## 2. Results

### 2.1. Literature Search and Eligible Studies

**Figure 1:**
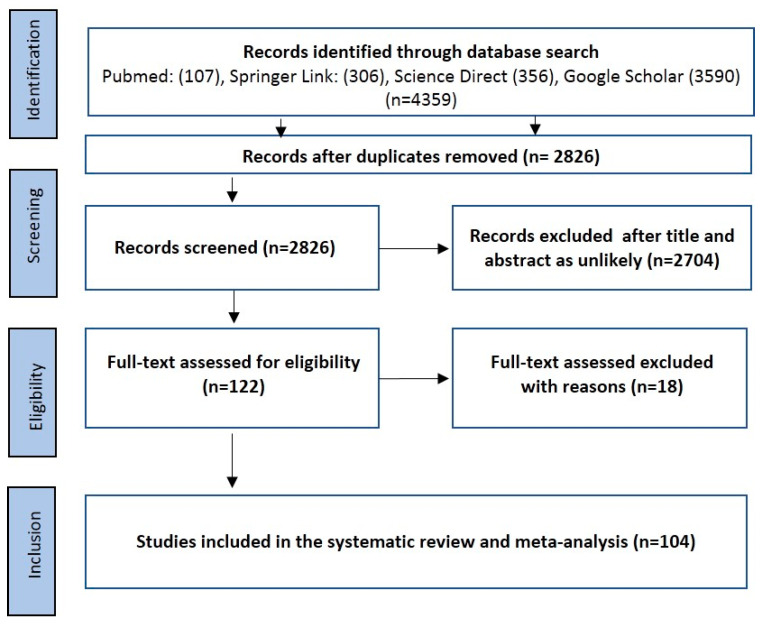
Preferred reporting items for systematic reviews and meta-analyses (PRISMA) flowchart used in the selection of eligible studies.

A total of 4359 relevant articles were identified following a search for all four databases using the procedure enumerated in Figure 1. After the removal of duplicates, we were left with 2826 studies for further review. A careful review of the titles and abstracts was done, and a total of 122 full-text articles were downloaded for detailed review. In total, 18 studies were excluded for various reasons. These included (i) the exact number of positive *Babesia* isolates were not clearly stated (*n* = 5), (ii) non-separation of the number of positive isolates of *Babesia* from questing ticks and other vertebrate hosts/feeding ticks (*n* = 4), (iii) incomplete information on tick collections (*n* = 3), (iv) lack of delineation of the results of positive *Babesia* species from other piroplasms (*n* = 2), and (v) no information on the number of tick DNA used for polymerase chain reaction (PCR) screening (*n* = 2), (vi) study with samples size below 40 (*n* = 2). One hundred and four (104) studies were further subjected to the quantitative synthesis. The quality assessment score (QAS) from the Joanna Briggs Institute (JBI) critical appraisal ranges from 6 to 8 out of a possible score of 9, equivalent to 66.7–88.89% in 100 out of the 104 included studies. Only 4 studies had a score of 5 (55.67%) (Table 1; Supplementary Table S2).

### 2.2. Characteristics of Eligible Studies

The characteristics of all eligible studies comprising of 137,364 ticks from 104 studies across different regions of the world are presented in Table 1. Included studies were from Europe (*n* = 78), North America (*n* = 13), Asia (*n* = 12), and Africa (*n* = 1). All eligible studies were carried out using molecular techniques to screen for tick-borne pathogens with particular reference to *Babesia* species. The prevalence for all the individual studies was computed and presented in Table 1. Individually, apart from a few studies, which recorded a 0% prevalence, the majority of the studies ranges from 0.25% to 12.96%, with a median of 1.78%. There were two studies with a prevalence of 20.65% and 21.67% and another two studies with a prevalence of 51.04% and 58.33% (Table 1). The majority of the studies were carried out from the year 2000 onward, with only one study undertaken in 1985.

### 2.3. Pooling, Heterogeneity and Subgroup Analysis

#### 2.3.1. Prevalence Based on Tick Species, Life Stages, Sex, and Diagnostic Technique

**Figure 2:**
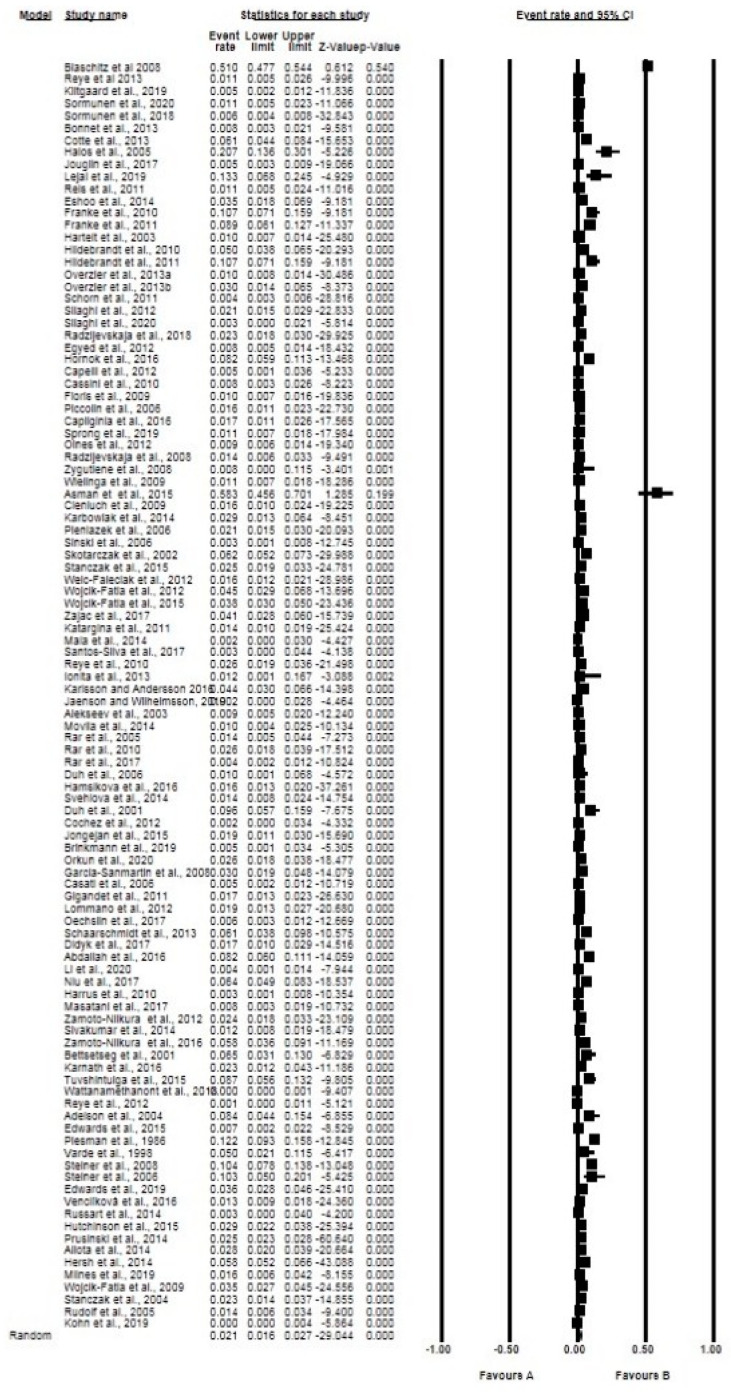
Forest plot showing the pooled prevalence of *Babesia* species globally. N.B. The squares show the individual point estimate. The diamond at the base indicate the pooled estimates from the total studies. Event rate: the frequency of occurrence of an event in a population, and it takes into account the possibility of an event occurring several times in an individual.

**Figure 3:**
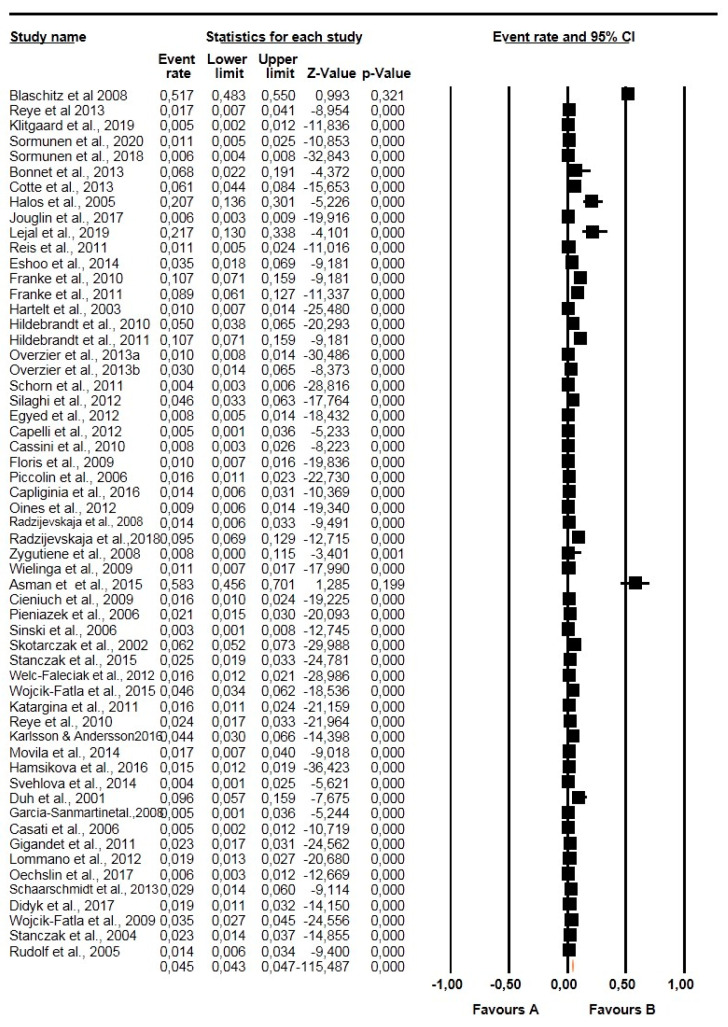
Forest plot showing the prevalence of *Babesia* species in questing *Ixodes ricinus* in Europe. N.B. The squares show the individual point estimate. The diamond at the base indicate the pooled estimates from the total studies. Event rate: is the frequency of occurrence of an event in a population, and it takes into account the possibility of an event occurring several times in an individual.

The overall and subgroup prevalence estimates of *Babesia* spp. based on tick species, life stages, sex and diagnostic technique, including confidence intervals and statistical parameters, are presented in Table 2. Globally, the overall pooled prevalence estimated (PPE) for *Babesia* species in questing ticks was 2.10% for all studies with 3069 positive cases from a total of 137,364 ticks screened and substantial study heterogeneity was observed (Table 2; Figure 2). *Babesia* species were detected in 23 different tick species within 4 genera *Ixodes* (5 species), *Dermacentor* (4 species), *Rhipicephalus* (4 species), *Haemaphysalis* (9 species) and *Hyalomma* (1 species) (Table 2). *Ixodes ricinus* was the most collected tick species with over 74,802 ticks in number and 1756 positive cases with PPE at 2.40% (Table 2; Figure 3). Other tick species included: *I. persulcatus* with PPE at 1.50%, *I. scapularis* at 4.10%, *D. reticulatus* at 2.10%, and *H. longicornis* at 4.30% (Table 2).

Other tick species that were reported, but no *Babesia* species were detected: *H.* sp. 1 & 2 [8]; *H. bispinosa* [28]; *Hy.* spp. [109]; *H*. *hystricis* and *H. kitaokai* [110]; *Amblyomma testudinarium* [110]; *I. nipponensis* [110]; *I. turdus* [37,110]; *I. tanuki* [37]; *H. douglasi* [29,37]; *H. megaspinosa* [29]; *H. wellingtoni* [112].

With regard to tick life stages, we observed an increasing infection rate from larvae with 0.60% to nymphs with 1.70% and the highest in adults with 2.60% (Table 2). Statistically significant differences (*p* < 0.0001) were observed across the different life stages. Additionally, the infection rate between the adult and larva was significantly different (*p* = 0.0033). The PPE was significantly (*p* = 0.0211) higher in the females with 4.90% compared to the males with 3.60% (Table 2).

**Figure 4:**
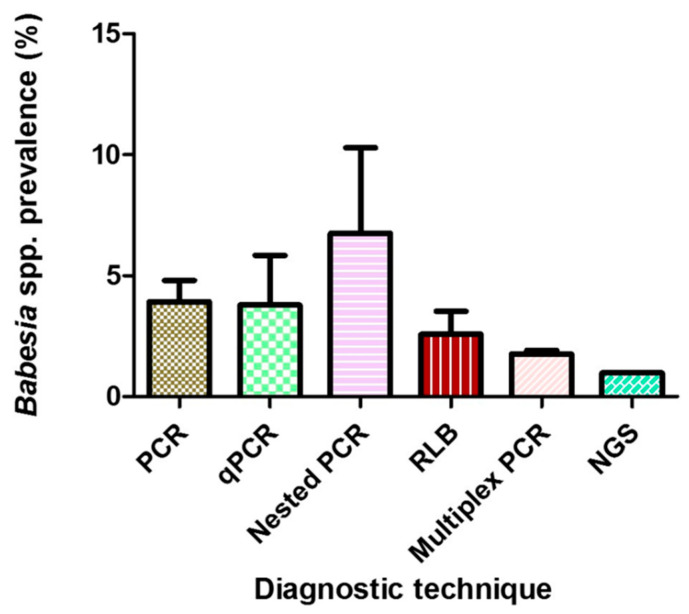
Mean prevalences of *Babesia* species globally using different diagnostic techniques. Error bars, standard errors of the means.

Six different molecular diagnostic techniques were employed in all the included studies, with conventional PCR being the most widely utilized in 66 studies with a PPE of 1.90%. Others include nested-PCR with 2.80% and qPCR with 1.70% (Table 2, Figure 4).

#### 2.3.2. Prevalence Based on *Babesia* Species, Region, and Sampling Periods

**Figure 5:**
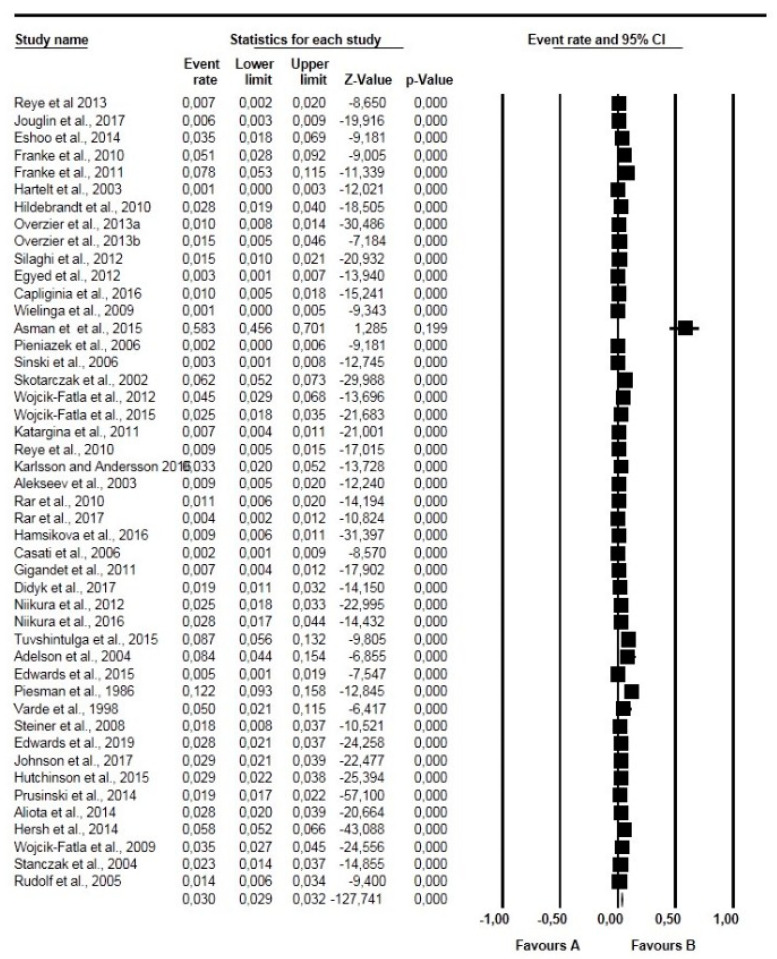
Forest plot showing the prevalence of *Babesia microti* globally. N.B. The squares show the individual point estimate. The diamond at the base indicate the pooled estimates from the total studies.

Globally, 19 different *Babesia* species were identified in ticks, with *B. microti* being the most observed species in 46 studies with a PPE of 1.90% (Table 3; Figure 5). This was followed by *B. venatorum* with 0.90% and *B. divergens* with 0.40%, which were exclusively found in ticks from Europe except for one study from Mongolia where *B. venatorum* DNA was amplified (Table 3). The prevalence of *B. ovata* was 0.60%, and *B.* spp. Xinjiang with 6.70% was observed only in ticks collected from Asia (Table 3).

According to region, Europe accounted for the majority of the studies (*n* = 78) with a PPE of 1.90% compared with Asia (*n* = 12) with a PPE of 2.00% (Table 3). North America had the highest PPE of 4.30% (Table 3). A single study was eligible from Africa, but none of the ticks was positive for *Babesia* spp.

We observed a statistically significant (*p* < 0.001) downward trend with respect to the PPE, with the highest being in period 1 (1992–1997) and the lowest in period 5 (2015–2020) (Table 3).

#### 2.3.3. Species Diversity of *Babesia* within Different Tick Species

**Figure 6:**
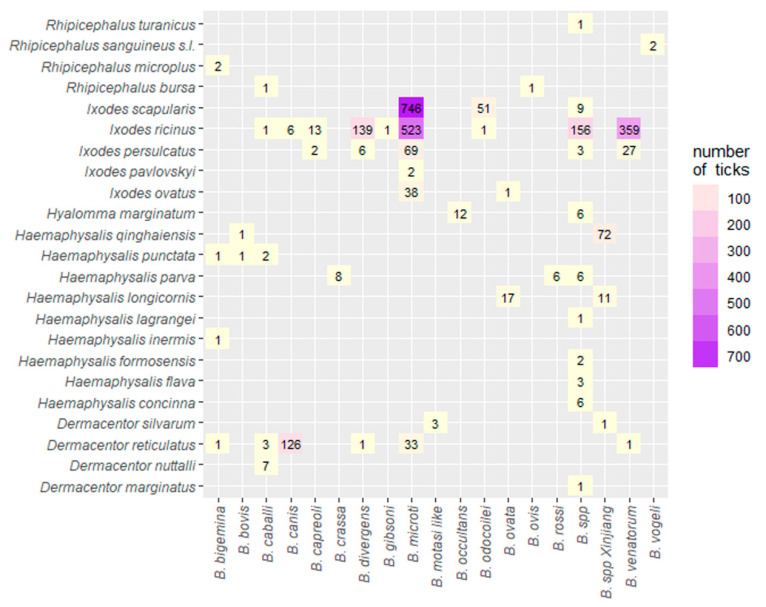
Heat map of the association of different *Babesia* species in different tick species globally.

The results of the distribution of different *Babesia* species according to the different tick species are presented in Figure 6. *Ixodes ricinus* was associated with 9 different *Babesia* spp. with *B. microti* and *B. venatorum* having the highest number of isolates: 523 and 359, respectively (Figure 6). Furthermore, *I. persulcatus* and *I. scapularis* ticks were associated with 5 and 3 different *Babesia* species, respectively, with a total of 911 *Babesia* isolates shared between both ticks. Additionally, *B. microti* accounted for 746 *Babesia* isolates in *I. scapularis*. Finally, *D. reticulatus* was associated with 6 different *Babesia* species, with *B. canis* being the highest with 126 isolates (Figure 6).

### 2.4. Spatial Distribution of Eligible Studies

**Figure 7:**
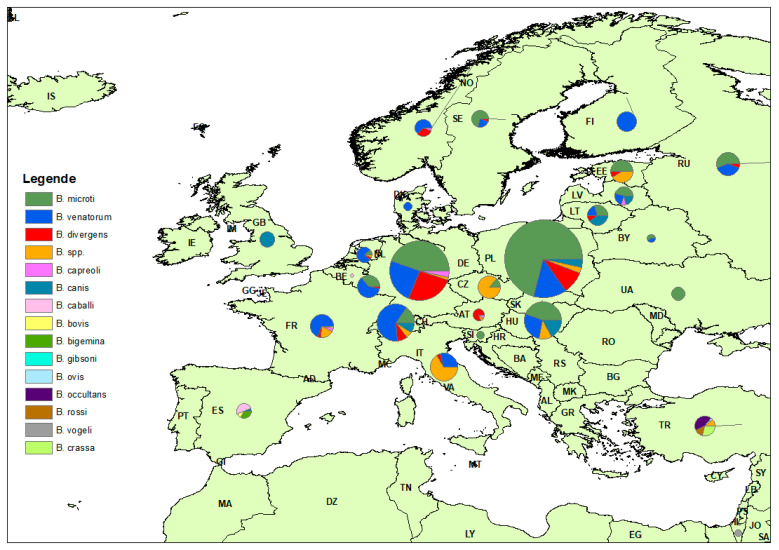
Distribution of *Babesia* species in different tick species across Europe.

In total, the results for 36 individual countries across four continents are presented in Table 4. In Europe, Poland and Germany had the highest number of eligible studies with 13 and 12 entries, each with PPE of 3.40% and 2.20%, respectively (Table 4). In addition, United States had 12 eligible studies with a PPE of 4.30%. Some other countries, including France, Russia, and Switzerland, have a PPE of 3.30%, 1.20% and 1.50%, respectively. A map with the spatial distribution of *Babesia* spp. across the different countries in Europe in different tick species is shown in Figure 7.

### 2.5. Publication Bias

The funnel plots and their corresponding bias coefficient (Begg and Mazumdar rank) for the estimation of the overall pooled MIR for published studies (*Z* = −48.00, *p* = 0.446) provides no evidence for the presence of publication bias among the eligible studies globally. For a few subgroup analyses, significant publication bias was observed for studies used for the computation of *B. canis* (*Z* = −35.00, *p* = 0.05), *B. divergens* (*Z* = −72.00, *p* = 0.01) and *B. microti* (*Z* = −203.00, *p* = 0.02). Additionally, mild bias was observed in studies from Asia (*Z* = −32.00, *p* = 0.014).

## 3. Discussion

### 3.1. Babesia Species in Ticks with Medical Importance

With the dawn of DNA-based techniques, molecular characterization has fostered the description and classification of new *Babesia* species. Therefore, the list of new species of *Babesia* continues to increase. In an attempt to synchronize the results from diverse epidemiological surveys for *Babesia* piroplasms in unfed host-seeking ticks comprising all live stages collected from vegetation across the globe, we undertook a systematic review and meta-analysis to estimate the pooled prevalence using random effect models.

Undoubtedly, *Babesia microti* was the most prevalent and widespread species of *Babesia* found in questing ticks in this study. DNA of *B. microti* has been detected in Europe, North America, and Asia with a PPE of 1.90%. This finding is comparable to the individual prevalence rates reported in previous studies [27,38,107,117]. Higher prevalence rates above 5.00% have also been reported in several other countries like United States [114,121], Poland [79,82] and Mongolia [32].

*Babesia microti*, *B. duncani*, *B. divergens* and *B. venatorum* are all regarded as zoonotic *Babesia* species. Clinically, most infected individuals are asymptomatic but could register lethal evolution depending on the species of *Babesia* and immunocompetence of the patient [18]. It is important to note that *B. microti* is responsible for most cases of human babesiosis and with great impact in North America but rare in Europe and Asia [18]. In Europe, both *B. divergens* and *B. venatorum* (formerly *Babesia* spp. EU1) are the predominant species causing human babesiosis. Interestingly, no study reported the detection of *B. duncani* in questing ticks. However, a recent report suggests the possible role of larval forms of *D. albipictus* as a possible vector of *B. duncani* transmission [122].

With the exception of one study from Mongolia [111], studies reporting the detection of *B. divergens* and *B. venatorum* were exclusively found in Europe with a PPE below 1.00%. This finding is comparable to the reports from over 70% of studies reporting the detection of this *Babesia* species in Europe [17,47,48,65,106]. The widespread presence of these species of *Babesia* of zoonotic importance in questing ticks has public health implications, especially in recreational parks during the period of tick activity. Therefore, humans could be exposed to pathogens with tick bites. Alternatively, blood transfusion-associated transmission has been reported in endemic areas, and it is regarded as the most common way of transmission in North America [123]. Therefore, Giemsa stained blood, serological testing or the use of PCR may significantly reduce the likelihood for transmission to occur by blood transfusion in endemic areas. Naturally, *B. microti* and *B. divergens* parasitize microtine rodents and cattle, respectively, these hosts being regarded as their reservoir [12]. On the other hand, *B. venatorum* is maintained naturally in wild cervids (deer), while the mule deer (*Odocoileu hemionus*) and possibly other species of wild ungulates in western North America may be the primary reservoir for *B. duncani* [122].

### 3.2. Babesia Species in Ticks with Veterinary Importance

Several species of *Babesia* are causing babesiosis in animals, including *B. bovis*, *B. bigemina*, *B. occultans*, *B. divergens*, *B. ovata*, *B. odocoilei* and *B. capreoli* (large ruminants and deer); *B. caballi* (equines); *B. crassa*, *B. ovis*, *B. motasi*-like and *B.* spp. Xinjiang (small ruminants), and *B. vogeli*, *B. canis*, *B. rossi* and *B. gibsoni* (canines). These species were observed in questing ticks across several regions. Of these species, some were observed to be geographically restricted (like *B. ovata* and *B.* spp. Xinjiang in Japan and China, respectively), in addition to uncharacterized *Babesia* species. The PPE for animal babesiosis in questing ticks ranges between 0.30% and 1.50%, with the exception of *B*. spp. Xinjiang with a PPE of 6.70%. These low prevalences are comparable to the infection rates reported for individual studies [24,29,41,43,47,68,108].

The PPE for *B. canis* was low, comparable to the prevalence reported in ticks from Slovakia [97], Russia [22] and Germany [24]. Furthermore, we observed that with the exception of *B. canis*, the agent of canine babesiosis, all other species of *Babesia* causing babesiosis in dogs were only reported separately, in one study each. Nonetheless, *B. canis* was reported in 14 studies from Europe. Therefore, *B. canis* appears to be the principal agent of canine babesiosis in Europe. In autochthonous cases where clinical canine babesiosis was reported, flagged ticks (*Dermacentor reticulatus*) in surrounding areas were positive to *B. canis* [23,41]. Additionally, in the majority of the studies (about 78%), *B. canis* DNA was reported in *D. reticulatus* tick, which is a competent vector for the protozoan parasite and is frequently found in urban biotypes in Europe [21].

*Babesia caballi*, one of the etiological agents of equine piroplasmosis, was observed at a low infection rate. The DNA of *B. caballi* was observed in *R. bursa* [44], *D. nutalli* [45] and *D. reticulatus* [41,44]. In the latter studies, both *B. caballi* and *B. canis* were detected in *D. reticulatus* ticks. Interestingly, both *B. canis* and *B. caballi* can be maintained for several generations in *D. reticulatus* ticks [41].

The PPE for agents of small ruminant’s babesiosis in questing ticks is consistent with reports from other individual studies where they occur at a very low prevalence [43,108]. Unlike *B. motasi* in Europe, *B.* spp. Xinjiang is known to principally infect sheep in China. From all available evidence, their presence in questing ticks is very low. Nonetheless, this *Babesia* spp. (*B*. spp. Xinjiang) has been amplified from blood samples from sheep and goats in China [28]. Earlier studies reported that *Hy anatolicum anatolicum* is the principal and competent vector [124]. The detection of *B*. spp. Xinjiang in *H. longicornis* and *H. qinghaiensis,* which are widespread in China, has raised several questions of their potential as vectors, but this remains speculative, and further studies will be required to verify this claim [28]. Additionally, *B. crassa* was detected in questing *H. parva* ticks in Turkey [108].

We observed seven species of bovine/cervid *Babesia* in host-seeking ticks. Unlike the virulent *B. bovis* and *B. bigemina*, *B. ovata* is of lower pathogenicity in cattle [29] and is one of the geographically restricted species of *Babesia*, similar to *B*. spp. Xinjiang in China. *Babesia ovata* is endemic in Japan and principally infects cattle [29]. *H. longicornis* is a known competent tick vector that can transmit the protozoan parasite transovarially [29,125], and further studies are needed to ascertain the probable role of *I. ovatus* that was observed to harbor this *Babesia* species. Additionally, *B. occultans* DNA was reported in *Hy. marginatum,* which is a known competent vector with empirical evidence from natural transovarial transmission [108], and transstadial survival [126]. Therefore, transstadial persistence of *B. occultans* in *Hy. marginatum* has been attributed to transovarial transmission of the pathogen [108], as only the adult ticks feed on the blood from cattle [127].

### 3.3. Ticks as Vectors of Babesia Species

*Ixodes ricinus* was the most abundant tick species in this study. This is not surprising considering that majority of the studies were from Europe, where this tick is predominant and a vector of several pathogens of protozoan, viral and bacterial agents of veterinary and medical importance [128,129]. Reports from various studies indicate that this tick is mostly found in urban and peri-urban areas in city parks, gardens, forest patches and litter layers [129]. Forested areas and particularly mixed and deciduous forests provide a sheltered canopy, and this tick species thrives due to the microclimates provided [128,130]. Due to climate change, current evidence points to the increasing distribution of *I. ricinus* steadily towards higher latitudes and altitudes. This was obvious in this study as several works were found investigating the presence of *Babesia* pathogen in questing tick in Sweden [92,93] and Finland [50]. Furthermore, *I. ricinus* harbors diverse *Babesia* species, which have been reported in Western Europe [46,52,73], Eastern Europe [98,99], Central Europe [39,104], Scandinavia [70,71,92], Southern Europe [44,64] and Balkan Peninsula [100], with varying prevalence and spread across the continent.

Other species within the genus *Ixodes,* such as *I. persulcatus*, *I. ovatus* and *I. pavlovskyi,* were reported in the Northern Hemisphere precisely in Russia and parts of southern Eurasia to harbor *Babesia* spp. at a prevalence ranging from 0.30 to 1.60%. According to [131], *I. persulcatus* ticks are closely related to *I. pavlovskyi*. For now, the vector competence of *I. pavlovskyi* is largely unknown. Nonetheless, *I. persulcatus* has been implicated as a possible competent vector for *B. divergens* [132].

*Ixodes scapularis* is widely distributed in the northeast, upper Midwest, mid-Atlantic and southeast states of the United States as well as in Canada [26,133] and was observed to be the major tick vector reported from North America. The PPE of *Babesia* spp. in this tick was low at 3.60%, comparable to the prevalence reported from other individual studies [118,120]. Higher prevalence has been reported in other parts of America [113,117]. Both *B. microti* and *B. odocoilei* are *Babesia* spp. found to be associated with this tick species causing human and cervid (white-tailed deer) babesiosis, respectively. The vector competence for *B. odocoilei* is unknown, but *I. scapularis* has been involved [42].

*Haemaphysalis longicornis* was reported in five studies, all from eastern Asia, where this tick species is native and originated from. The PPE was low to moderate at 4.3%. This tick species was observed to harbor *B. ovata* in Japan [29] and *B*. spp. Xinjiang in China [28]. Therefore, babesiosis in cattle and sheep, respectively, in that region is believed to be caused by *B. ovata* and *B.* spp. Xinjiang is probably transmitted by *H. longicornis*.

### 3.4. Association between Ticks and Babesia Including Other Factors

Ixodid tick species play a crucial role in the epidemiology of babesiosis. Reports of the detection of *Babesia* DNA may not necessarily denote evidence of vector competence, whether in unfed or engorged ticks [134]. In transovarial transmission, most *Babesia* species invade the tick ovaries and persist in the larvae. Consequently, infection is transmitted vertically. The acquisition of the parasites (*Babesia* species) from their respective host by either the larvae or nymphs is referred to as transstadial transmission.

Furthermore, of all tick species in this study, *I. ricinus* had the highest association with several *Babesia* species with three and six species of human and veterinary importance, respectively. This tick is a known competent vector for 3 *Babesia* parasites (*B. divergens*, *B. venatorum* and *B. microti*), causing human babesiosis [129]. Since all stages (larvae, nymph, and adult) of *I. ricinus* can transmit B. *divergens* and *B. venatorum*, the risk of infection is high after tick bites in humans during periods of peak tick activity. Detailed review on the association of *I. ricinus* with *Babesia* and other tick-borne pathogens can be obtained elsewhere [129,134].

The fact that the adult ticks and, by extension, female ticks were the most predominant with the highest infection rates compared with the nymphs and larvae may have some implications in transmission. In transovarial transmission involving most *Babesia* species, it has been asserted that only the female ticks can acquire the infection. Immature stages are less likely to become infected due to the smaller blood volumes they ingest. Furthermore, the fewer number and size of the midgut epithelial basophilic cells of immature stages, which play a role in parasite development, are believed to be an important factor as well [134]. Furthermore, evidence of transstadial transmission has been observed for some *Babesia* spp., but also, not all tick stages are capable of transmitting the parasite as observed for *B. bovis*, where only the larvae of *R. annulatus* can transmit. On the other hand, only the nymphal and adult stages of *R. annulatus* can transmit *B. bigemina* [134]. Additionally, many *Babesia* spp., including *B. major*, *B. motasi*, *B. rossi*, *B. venatorum*, *B. vogeli* and *B. divergens,* can persist from larval to their adult stages (transstadial transmission) in their competent vectors without reinfection for a minimum of one generation [134].

Female ticks had higher infection rates compared with their male counterparts. It is well known that female ticks require blood meals to develop their ovaries and lay thousands of eggs to perpetuate their existence. In addition, as earlier mentioned, the transovarial transmission is one of the utmost successful evolutionary strategies among the Apicomplexa and specifically in *Babesia* sensu stricto [20]. Therefore, female ticks take larger blood meals (high volume of blood) due to prolonged feeding, which may result in higher chances of infection. Furthermore, females require a higher number of blood meals for molting before reaching the adult stage.

The use of molecular-based techniques for the diagnosis and classification of *Babesia* species has been widely adopted due to greater sensitivity and specificity [18]. All studies used molecular-based techniques. In the various epidemiological investigation of *Babesia* species in questing ticks as observed in this study, several molecular approaches, including qPCR [49,120], nested-PCR [8,28,74], conventional PCR [47,57,88], reverse line blot hybridization [39,43] and more recently, next-generation sequencing [33] among other methods have been adopted. Despite the observation of differences in the prevalence rates between techniques, no statistical significance was noted. Similar findings were observed in a Euro-wide meta-analysis of *Borrelia burgdorferi* sensu lato prevalence in questing *I. ricinus* ticks [135]. The highest in the prevalence rate was nested-PCR, but it is difficult to conclude considering the fact that the number of studies that utilized this technique is comparatively fewer compared with the conventional PCR. The geospatial distribution indicates that extensive studies have been conducted in Germany, Poland, and United States. This observation could be connected with a research interest in those countries with a bias towards tick-borne diseases.

This systematic review has spawned data on the prevalence of *Babesia* species in questing ticks. However, some limitations were observed in our study. First, we excluded articles published in languages other than English, and hence some vital information may have been set aside. Second, our study focused only on questing ticks; therefore, areas without reported *Babesia* pathogen may still have the pathogen. Third, due to the use of different DNA-based techniques with varying sensitivity, some *Babesia* species with low detection sensitivity might have been missed. Fourth, the global prevalence was obtained from studies from four continents. Therefore, the global pooled prevalence of *Babesia* spp. may vary from the actual estimate, but we believe that the apparent prevalence in this study is close to normal. Fifth, the heterogeneity observed could be due to sampling error, sample size, or variation of endemicity and study design. Despite the limitations highlighted above, this study used a large number of eligible studies (n = 104) and ticks screened (137,364) from a global perspective to clearly provide a comprehensive insight and meta-analysis on the distribution of *Babesia* species in different questing ticks across four continents from published literature. Our results clearly indicate that these ticks harbor potentially disease-causing *Babesia* parasites of human and veterinary importance.

## 4. Material and Methods

### 4.1. Search Strategy

We followed the protocol as outlined by the preferred reporting items for systematic reviews and meta-analyses (PRISMA) in carrying out this systematic review and meta-analysis [136]. We searched for citations with no time restrictions through to 10 July 2020 solely in English databases of Science Direct, Springer Link, PubMed, and Google Scholar. Key operators used in the systematic search were “*Babesia*”, “questing ticks”, and “tick-borne pathogens”. Key terms used in the search were used individually or in combination with “AND” and/or “OR” operators. Duplicates were removed, and relevant titles and abstracts were scanned, and those articles in line with the aim of the study were downloaded.

### 4.2. Inclusion and Exclusion Criteria

Selected relevant articles, after the review of titles and abstract, were downloaded for further screening of the full text for eligibility. Included articles for the study must fulfill the following seven criteria, namely (i) the collected ticks must be questing ticks from vegetation, (ii) the total number of ticks screened was stated, (iii) the country of the study was known, (iv) the study screened for the presence of *Babesia* in questing ticks, and the number of positives/negatives was stated (v) the molecular diagnostic method employed in the study was stated (vi) for a tick species to be included in the result, at least one *Babesia* spp. DNA must have been amplified for that species (vii) no limit to the minimum sample size of screened ticks, but for statistical reasons, it was set at less than 40 samples. Where the exact number of the respective live stages were not clearly stated, the total number of screened ticks collected for that study was used only in the computation of the overall prevalence. Studies were excluded if (i) the exact number of positive *Babesia* isolates were not clearly stated, (ii) separation of the number of positive isolates of *Babesia* from questing ticks and other vertebrate host/feeding ticks was missing, (iii) incomplete information on tick collections (iv) lack of delineation of the results of positive *Babesia* species from other piroplasms (v) no information on the number of tick DNA samples used for PCR screening (vi) study with sample size below 40.

### 4.3. Data Cleaning

In most of the studies, the developmental stages (larva and nymphs) were pooled before pathogen detection. Therefore, we calculated the minimum infection rates (MIR) (based on the assumption of a single positive tick per pool) for all included studies to avoid overestimation of a prevalence. Consequently, the prevalence throughout reflects the MIR in ticks. With regard to the years of sampling, where sampling was undertaken over two or more years, and the results were presented separately for each year, we divided the entries accordingly. Similarly, where entries involved different tick species and countries but published on the same articles, the data were separated meticulously. For the calculation of the overall prevalence, we used data from all eligible studies incorporating the total number of ticks screened irrespective of the live stages. Overall, only tick species that showed at least one positivity to *Babesia* spp. were presented in Table 2. Therefore, tick species reported without any single cumulative positivity to *Babesia* spp. were not included in the results. Furthermore, the number of positive *Babesia* spp. isolates that were confirmed by good quality sequences as reported in the articles were used for the subgroup analysis (*Babesia* species).

### 4.4. Data Extraction

All studies meeting the inclusion criteria were cataloged, and data were extracted using a charting form developed by the research team. Data extracted from all the eligible studies included all the variables as contained in the inclusion criteria, such as the name of the authors, year of sampling, geographical location, the total number of ticks screened, the molecular diagnostic technique used, the life stages of the ticks, tick species, sex of the ticks, species of *Babesia* detected as well as the number of positive/negative *Babesia* isolates. The MIR was calculated according to the various subgroups.

### 4.5. Quality Assessment of Included Studies

The quality assessment of each article included in the study was undertaken using the Joanna Briggs Institute (JBI) critical appraisal instrument for studies with prevalence data [137]. This JBI instrument consists of nine questions, of which details are available (Supplementary Table S1). Each answer to the individual question was assigned a score of 0 or 1 for no or yes answers. When the question was not applicable to the study, not applicable (NA) was used. Results of *Babesia* species distribution were summarized on a country level and exported as a CSV file into ArcGis Desktop (Esri, version 10.5.1, Redlands, CA, USA). Data were visualized in pie charts per country.

### 4.6. Statistical Analysis

All statistical analyses were carried out using Comprehensive Meta-analysis (CMA) Version 3.0 by Biostat (Englewood, NJ, USA) unless otherwise stated. The weighted pooled minimum infection rate (MIR) and 95% confidence interval (CI) were computed. For each individual study, we recalculated the MIR (prevalence) by summing the total number of samples and positive cases irrespective of the number of tick species reported for that study. When the pooled analysis was performed, each logit event estimate undergoes a transformation within the CMA software into proportions with its corresponding 95% CI. We calculated the overall MIR as a percentage. Forest plots were used to visualize the data generated. Cochran’s heterogeneity (*Q*) among the included studies, as well as the percentage inverse variation (I^2^), was calculated using the Cochrane *Q* test. If I^2^ was ≤25%, 50% or ≥75%, then heterogeneity was described as low, moderate, or high (substantial), respectively [138]. If there was only a single study for a particular category, the positive rate was computed without heterogeneity (*Q*). All pooled estimates were arrived at using a random-effects model except for sex, where we used the fixed-effect model due to the homogeneity of the data. The chi-squared test was used to test for significance for all the subgroups using GraphPad Prism, version 5.04 (GraphPad Software, Inc, La Jolla, CA, USA, www.graphpad.com). *p* values of <0.05 were considered statistically significant unless otherwise stated. Funnel plots using visual inspection and the Beg and Mazumdar rank correlation test [139] were used for assessing the publication bias.

## 5. Conclusions

In this meta-analysis of pooled data on *Babesia* species in questing ticks from a global perspective, our findings indicate both human and animal *Babesia* species DNA in a variety of species of questing hard ticks with low to moderate prevalence. We reported the detection of 19 *Babesia* species in 23 different tick species across four continents. Adult male and female ticks had the highest infection rates compared with immature and male ticks, respectively. *Ixodes ricinus* was the main tick species of interest, and it is a tick species of economic importance, with *B. microti* being the most widely detected species of *Babesia* across the different regions. The information generated from this study will be helpful to the relevant stakeholders in the design and future implementation of programs aimed at controlling competent vectors against *Babesia* parasites.

## Figures and Tables

**Table 1:** Characteristics of all 104 studies used in the meta-analysis of molecular *Babesia* detection in questing ticks.

Study Year	Country	Continent	Molecular Technique	Sample Size	Cases	MIR	JBI QAS	Study Ref.
2005	Austria	Europe	PCR	864	441	51.04	5	[2]
2009	Belarus	Europe	PCR	453	5	1.10	7	[48]
2016–2017	Denmark	Europe	qPCR	1013	5	0.49	7	[49]
2015	Finland	Europe	qPCR/PCR	515	6	1.17	7	[50]
2012–2017	Finland	Europe	qPCR	7070	41	0.58	8	[51]
2009	France	Europe	PCR/RLB	495	4	0.81	7	[52]
2006–2007	France	Europe	PCR	572	35	6.12	8	[53]
2002	France	Europe	PCR	92	19	20.65	8	[54]
2012–2013	France	Europe	PCR	2620	15	0.57	8	[55]
2017	France	Europe	qPCR	60	8	13.33	8	[56]
2008	France	Europe	PCR	558	6	1.08	8	[40]
2009	Germany	Europe	PCR	226	8	3.54	8	[57]
2007	Germany	Europe	PCR	196	21	10.71	8	[36]
2008	Germany	Europe	PCR	293	26	8.87	8	[58]
1999–2001	Germany	Europe	PCR	3113	31	0.99	8	[59]
2006–2007	Germany	Europe	PCR	1000	50	5.00	8	[60]
2006	Germany	Europe	PCR	196	21	10.71	8	[61]
2011–2012	Germany	Europe	PCR	4381	45	1.03	8	[46]
2011	Germany	Europe	PCR	199	6	3.02	8	[38]
2009–2010	Germany	Europe	PCR	6593	28	0.42	8	[47]
2008–2010	Germany	Europe	PCR	1721	36	2.09	8	[17]
2010–2013	Germany	Europe	PCR	339	1	0.29	8	[24]
2011–2012	Germany	Europe	PCR	2000	0	0	8	[62]
* 2010–2018	Germany, Netherland, Belgium, and Great Britain	Europe	Microfluidic qPCR	1486	16	1.08	6	[25]
2006–2008	Hungary	Europe	PCR	1800	15	0.83	5	[63]
2014–2015	Hungary	Europe	PCR	413	34	8.23	8	[21]
2006–2008	Italy	Europe	PCR	191	1	0.52	8	[64]
2006	Italy	Europe	PCR	356	3	0.84	6	[65]
2006–2007	Italy	Europe	Nested PCR	1861	19	1.02	7	[66]
2000–2001	Italy	Europe	Multiplex PCR	1931	31	1.61	5	[67]
2005–2007	Latvia	Europe	PCR	1125	19	1.69	8	[68]
2006	Latvia and Lithuania	Europe	Nested PCR	2810	40	1.42	8	[69]
2006–2008	Norway	Europe	qPCR, nested PCR	1908	17	0.89	8	[70]
2006	Norway and Lithuania	Europe	qPCR	364	5	1.37	8	[71]
2005	Lithuania	Europe	PCR	62	0	0	7	[72]
2003–2007	Netherlands	Europe	RLB/PCR	1488	16	1.08	7	[73]
NA	Poland	Europe	Nested PCR	60	35	58.33	6	[74]
2008	Poland	Europe	Nested PCR	1392	22	1.58	8	[75]
2009–2012	Poland	Europe	Nested PCR	205	6	2.93	5	[76]
2001	Poland	Europe	PCR	1328	28	2.11	7	[77]
2000–2004	Poland	Europe	PCR	1513	5	0.33	8	[78]
1999	Poland	Europe	PCR	2095	130	6.21	8	[79]
2009–2010	Poland	Europe	qPCR	1875	47	2.51	8	[80]
2009–2010	Poland	Europe	PCR	3165	50	1.58	6	[81]
2008–2009	Poland	Europe	PCR, nested PCR	468	21	4.49	8	[82]
2011–2012	Poland	Europe	PCR, nested PCR	1435	55	3.83	8	[83]
2011	Poland	Europe	PCR	634	26	4.10	7	[84]
2004–2006	Poland	Europe	Nested PCR	1620	57	3.52	7	[85]
2001	Poland	Europe	Nested PCR	701	16	2.28	7	[86]
2006–2008	Estonia	Europe	RLB, nested PCR	2603	36	1.38	6	[87]
2012	Portugal	Europe	PCR	263	0	0.0	8	[88]
2012–2013	Portugal	Europe	PCR	175	0	0.0	8	[89]
2007	Luxembourg	Europe	PCR	1394	36	2.58	7	[90]
2010	Romania	Europe	PCR	40	0	0	8	[91]
2013–2014	Sweden	Europe	PCR	519	23	4.43	7	[92]
2015–2016	Sweden	Europe	PCR	277	0	0	8	[93]
2000	Russia	Europe	PCR	738	7	0.95	6	[94]
2009	Russia	Europe	PCR	481	5	1.04	6	[95]
2003–2004	Russia	Europe	Nested PCR	209	3	1.44	6	[22]
2008–2009	Russia	Europe	Nested PCR	922	24	2.60	6	[96]
2010–2015	Russia	Europe	Nested PCR	911	4	0.44	6	[31]
2002	Slovakia	Europe	PCR	100	1	1.0	8	[97]
2011	Slovakia	Europe	PCR	5148	78	1.63	8	[98]
2011–2012	Slovakia	Europe	PCR	886	12	1.35	7	[99]
1997	Slovenia	Europe	PCR	135	13	9.63	7	[100]
2003	Czech Republic	Europe	PCR	350	5	1.43	8	[101]
2011–2014	Czech Republic	Europe	PCR	2473	32	1.29	8	[102]
1997	Belgium	Europe	PCR	230	0	0	6	[103]
2011–2013	Netherlands and Belgium	Europe	RLB/PCR	855	17	1.99	7	[41]
2003–2005	Spain	Europe	RLB/PCR	562	17	3.03	8	[44]
2002–2003	Switzerland	Europe	PCR	865	4	0.46	7	[104]
2006	Switzerland	Europe	RLB/PCR	2568	44	1.71	8	[105]
2009–2010	Switzerland	Europe	RLB/PCR	1476	28	1.89	7	[39]
2015–2016	Switzerland	Europe	qPCR	1079	6	0.56	8	[106]
2012	Switzerland	Europe	PCR	261	16	6.13	8	[23]
2013–2014	Ukraine	Europe	PCR	767	13	1.69	7	[107]
2011–2013	Turkey	Europe-Asia	NGS	205	1	0.49	7	[33]
2014–2018	Turkey	Europe-Asia	PCR	1019	27	2.65	8	[108]
2013–2014	China	Asia	RLB/PCR	450	37	8.22	8	[43]
2013–2014	China	Asia	Nested PCR	558	2	0.36	8	[8]
2013–2014	China	Asia	Nested PCR	797	51	6.39	7	[28]
2013–2014	Israel	Asia	PCR	1196	3	0.25	6	[109]
2013–2015	Japan	Asia	Nested PCR	624	5	0.80	8	[110]
2000–2003	Japan	Asia	Nested PCR	1656	40	2.42	8	[37]
2008	Japan	Asia	PCR	1459	18	1.23	8	[29]
2000–2003	Japan	Asia	PCR	294	17	5.78	8	[30]
NA	Mongolia	Asia	Nested PCR	108	7	6.48	6	[45]
2009	Mongolia	Asia	PCR	400	9	2.25	8	[111]
2012–2013	Mongolia	Asia	Nested PCR	219	19	8.68	7	[32]
2015	Thailand	Asia	PCR	12,184	1	0.01	8	[112]
2009	Nigeria	Africa	PCR	700	0	0	8	[34]
2001	United States of America	North America	PCR	107	9	8.41	6	[113]
2013–2014	United States of America	North America	PCR	423	3	0.71	6	[114]
1985	United States of America	North America	PCR	395	48	12.15	8	[115]
1996	United States of America	North America	PCR	100	5	5.0	6	[116]
2003–2006	United States of America	North America	PCR	394	41	10.41	7	[117]
2003	United States of America	North America	PCR	68	7	10.29	6	[42]
2015–2017	United States of America	North America	HRM	1721	62	3.60	8	[118]
2010	United States of America	North America	PCR	191	0	0	8	[119]
2012–2014	United States of America	North America	qPCR	1855	54	2.91	8	[120]
2003–2004	United States of America	North America	Multiplex PCR	11,184	283	2.53	8	[27]
2011	United States of America	North America	PCR	1245	35	2.81	7	[35]
2011	United States of America	North America	qPCR	4368	255	5.84	8	[121]
2016–2017	Canada	North America	PCR	249	4	1.61	8	[26]

PCR: polymerase chain reaction; qPCR: real-time polymerase chain reaction; RLB: reverse line blotting; HRM: high-resolution melting; NGS: next-generation sequencing; NA: not available; MIR: minimum infection rate; JBI: Joanna Briggs Institute; QAS: quality assessment score. * Sprong et al. (2019): The sample number and results from Germany were excluded from our computation.

**Table 2:** Pooled minimum infection rate (MIR) estimates of *Babesia* spp. in questing ticks based on tick species, life stages, sex, and diagnostic technique.

Subgroup	Number of Studies	Pooled Prevalence Estimates			Measure of Heterogeneity		
		Sample Size	No of Positives	Weighted MIR95% CI (%)	*Q* Value	I^2^	*Q*−*p*
**All studies**	104	137,364	3069	2.10 (1.60–2.70)	4438.97	97.65	*p* < 0.0001
**Tick species**							
*Ixodes ricinus*	57	74,802	1756	2.40 (1.50–3.60)	3737.86	98.50	*p* < 0.0001
*I. persulcatus*	14	5823	102	1.50 (0.70–3.20)	154.44	91.58	*p* < 0.0001
*I. ovatus*	3	1420	39	0.60 (0.00–9.20)	17.23	88.39	*p* < 0.0001
*I. scapularis*	14	22,694	786	4.10 (2.70–6.20)	296.36	95.95	*p* < 0.0001
*I. pavlovskyi*	1	577	2	0.30 (0.01–1.40)	−	−	−
*Dermacentor* *reticulatus*	20	11,802	197	2.10 (1.30–3.50)	174.89	89.14	*p* < 0.0001
*D. marginatus*	2	390	1	0.80 (0.10–9.4)	2.26	55.65	*p* < 0.0001
*D. nuttalli*	3	389	7	1.30 (0.10–12.10)	7.60	73.76	*p* = 0.022
*D. silvarum*	2	223	4	1.80 (0.20–18.50)	3.06	67.23	*p* = 0.080
*R. bursa*	4	120	2	2.90 (0.90–8.50)	0.99	0.00	*p* = 0.802
*R. sanguineus s.l.*	5	1668	3	0.60 (0.10–2.60)	8.77	54.39	*p* < 0.001
*R. (Boophilus) microplus*	3	1498	2	0.30 (0.10–1.90)	1.63	0.00	*p* = 0.443
*R. turanicus*	1	9	1	11.1 (1.50–50.00)	0.00	0.00	*p* = 1.000
*Hemaphysalis* *longicornis*	5	626	28	4.30 (1.60–10.90)	13.17	69.62	*p* = 0.010
*H. concinna*	4	130	6	6.10 (3.00–11.90)	0.760	0.00	*p* = 0.825
*H. qinghaiensis*	2	430	73	17.20 (10.90–26.0)	4.32	76.86	*p* = 0.038
*H. punctata*	1	111	4	3.60 (1.40–9.20)	0.00	0.00	*p* = 1.000
*H. parva*	1	793	13	1.60 (1.00–2.80)	0.00	0.00	*p* = 1.000
*H. inermis*	1	87	1	1.10 (0.20–7.70)	0.00	0.00	*p* = 1.000
*H. flava*	2	282	3	1.30 (0.50–3.80)	0.49	-	*p* = 0.484
*H. formosensis*	1	159	2	1.30 (0.30–4.90)	0.00	0.00	*p* = 1.000
*H. lagrangei*	1	11,309	1	0.00 (0.00–0.01)	0.00	0.00	*p* = 1.000
*Hyalomma marginatum*	1	105	13	12.38 (7.30–20.20)	0.00	0.00	*p* = 1.000
**Life stages**							
Adult	79	55,411	1484	2.60 (2.00–3.40)	1693.34	95.34	*p* < 0.0001
Nymphs	53	44,746	1066	1.70 (1.10–2.50)	1578.82	96.77	*p* < 0.0001
Larvae	13	20,866	174	0.60 (0.10–3.60)	699.77	98.29	*p* < 0.0001
Sex							
Male	26	7534	199	3.60 (3.10–4.20)	145.53	82.82	*p* < 0.0001
Female	26	8395	275	4.90 (4.40–5.60)	256.98	90.27	*p* < 0.0001
**Diagnostic technique**							
Conventional PCR	66	76,021	1663	1.90 (1.30–2.90)	3339.99	98.05	*p* < 0.0001
qPCR	12	23,314	522	1.70 (1.00–3.00)	332.86	96.69	*p* < 0.0001
Nested PCR	16	14,653	376	2.80 (1.70–4.70)	339.97	95.59	*p* < 0.0001
RLB	7	10,002	195	2.20 (1.30–3.80)	85.88	92.99	*p* < 0.0001
Multiplex PCR	2	13,115	246	1.90 (1.70–2.10)	0.89	0.00	*p* = 0.344
NGS	1	205	2	1.00 (0.20–3.80)	0.00	0.00	*p* = 1.000

PCR: polymerase chain reaction; qPCR: real-time polymerase chain reaction; RLB: reverse line blotting; NGS: next-generation sequencing; I^2^: inverse variance; *Q*-*p*: Cochran’s; CI: confidence interval; MIR: minimum infection rate. Measure of heterogeneity: the weighted sum of squared differences between individual study effects and the pooled effect across studies.

**Table 3:** Pooled MIR estimates of *Babesia* in questing ticks based on *Babesia* species, region, and sampling periods.

Subgroup	Number of Studies	Pooled Prevalence Estimates			Measure of Heterogeneity		
		Sample Size	No of Positives	Weighted MIR95% CI (%)	*Q* Value	I^2^	*Q*−*p*
**All studies**	104	137,364	3069	2.10 (1.60–2.70)	4438.41	97.68	*p* < 0.0001
***Babesia*** **species**							
*Babesia microti*	46	68,537	1425	1.90 (1.40–2.50)	1071.94	95.80	*p* < 0.0001
*B. venatorum*	31	50,611	370	0.90 (0.70–1.10)	163.47	81.65	*p* < 0.0001
*B. divergens*	20	33,517	141	0.40 (0.20–0.70)	161.75	88.47	*p* < 0.0001
*B.* spp.	19	38,125	183	0.50 (0.20–1.10)	363.19	95.59	*p* < 0.0001
*B. capreoli*	6	15,927	13	0.10 (0.10–0.20)	7.31	31.61	*p* = 0.199
*B. canis*	15	14,938	132	1.10 (0.50–2.40)	235.83	94.06	*p* < 0.0001
*B. odocoilei*	6	8002	52	0.90 (0.20–4.50)	102.06	95.10	*p* < 0.0001
*B. caballi*	3	1525	17	1.40 (0.30–6.90)	20.02	90.01	*p* < 0.0001
*B. bovis*	2	1012	3	0.30 (0.10–0.90)	0.15	0.00	*p* = 0.700
*B. bigemina*	3	1570	7	0.50 (0.20–1.40)	2.75	27.16	*p* = 0.253
*B. ovata*	2	1909	18	0.60 (0.10–5.00)	2.85	64.87	*p* = 0.092
*B.* spp. *Xinjiang*	2	1247	84	6.70 (5.50–8.30)	0.39	0.00	*p* = 0.528
*B. gibsoni*	1	6593	1	0.00 (0.00–0.10)	0.00	0.00	*p* = 1.000
*B. ovis*	1	205	1	0.50 (0.10–3.40)	0.00	0.00	*p* = 1.000
*B. occultans*	1	1019	12	1.20 (0.70–2.10)	−	−	−
*B. rossi*	1	1019	4	0.40 (0.10–1.00)	0.00	0.00	*p* = 1.000
*B. vogeli*	1	1196	3	1.50 (0.00–32.40)			
*B. crassa*	1	1019	8	0.80 (0.40–1.60)	0.00	0.00	*p* = 1.000
*B. motasi* like	1	450	3	0.70 (0.20–2.00)	0.00	0.00	*p* = 1.000
**Region**							
Europe	78	94,376	2056	1.90 (1.30–2.70)	3964.12	98.06	*p* < 0.0001
Asia	12	19,945	209	2.00 (1.10–3.50)	174.67.69	93.70	*p* < 0.0001
North America	13	22,299	806	4.30 (3.00–6.20)	237.73	94.95	*p* < 0.0001
**Sampling period**							
1992–1997 (period 1)	3	465	18	4.30 (1.30–13.90)	8.28	75.85	*p* = 0.016
1998–2002 (period 2)	9	10,205	269	2.90 (1.40–5.70)	205.79	96.11	*p* < 0.0001
2003–2008 (period 3)	29	39,266	1326	2.60 (1.40–4.80)	2628.50	98.94	*p* < 0.0001
2009–2014 (period 4)	38	52,571	950	1.60 (1.20–2.20)	627.33	94.10	*p* < 0.0001
2015–2020 (period 5)	10	20,722	103	0.90 (0.40–2.10)	112.84	92.91	*p* < 0.0001

I^2^: inverse variance; *Q*-*p*: Cochran’s; CI: confidence interval; MIR: minimum infection rate. Measure of heterogeneity: the weighted sum of squared differences between individual study effects and the pooled effect across studies.

**Table 4:** Prevalence estimates of *Babesia* in questing ticks based on country.

Subgroup	Number of Studies	Pooled Prevalence Estimates			Measure of Heterogeneity		
		Sample Size	No of Positives	Weighted MIR95% CI (%)	*Q* Value	I^2^	*Q*−*p*
Austria	1	864	441	51.00 (47.70–54.40)	−	−	−
Belarus	1	453	5	1.10 (0.50–2.60)	−	−	−
Denmark	1	1013	5	0.50 (0.20–1.20)	−	−	−
Finland	2	7585	47	0.70 (0.40–1.40)	2.56	60.97	*p* = 0.109
France	6	4397	87	3.30 (0.90–10.80)	148.22	96.62	*p* < 0.0001
Germany	12	20,257	273	2.20 (1.10–4.40)	326.82	96.63	*p* < 0.0001
Hungary	2	2213	49	2.70 (0.30–22.0)	56.48	98.23	*p* < 0.0001
Italy	4	4339	54	1.20 (0.90–1.70)	3.93	23.64	*p* = 0.269
Latvia	2	1306	24	1.90 (1.30–2.80)	0.98	0.00	*p* = 0.323
Norway	2	2132	19	0.90 (0.60–1.40)	0.00	0.00	*p* = 0.998
Lithuania	3	2831	64	2.30 (1.80–2.90)	0.59	0.00	*p* = 0.042
Netherland	3	2893	32	1.20 (0.40–3.50)	13.34	85.01	*p* = 0.000
Poland	13	16,491	498	3.40 (2.10–5.50)	330.43	96.37	*p* < 0.0001
Estonia	1	2603	36	1.40 (1.00–1.90)	−	−	−
Portugal	2	438	0	0.02 (0.00–1.60)	0.041	0.00	*p* = 0.839
Luxembourg	1	1394	36	2.60 (1.90–3.60)	0.00	0.00	−
Romania	1	40	0	1.20 (0.10–16.70)	−	−	−
Sweden	2	796	23	1.20 (0.10–22.0)	5.14	80.53	*p* = 0.023
Russia	5	3261	43	1.20 (0.60–2.30)	15.48	74.15	*p* = 0.004
Slovakia	3	6130	97	1.60 (1.30–1.90)	0.57	0.00	*p* = 0.751
Slovenia	1	135	13	7.40 (4.00–13.20)	−	−	−
Czech Republic	2	2823	37	1.30 (1.00–1.80)	0.04	0.00	*p* = 0.836
Belgium	3	1053	1	0.20 (0.10–0.90)	0.54	0.00	*p* = 0.761
Britain	1	113	16	14.20 (8.90–21.90)	−	−	−
Turkey	2	1224	28	2.00 (0.80–4.80)	1.90	47.41	*p* = 0.168
Spain	1	562	17	3.00 (1.90–4.80)	−	−	−
Switzerland	5	6259	98	1.50 (0.80–3.00)	40.02	90.00	*p* < 0.0001
Ukraine	1	767	13	1.90 (1.10–3.20)	−	−	−
China	3	1805	90	4.10 (1.90–9.0)	19.63	89.81	*p* < 0.0001
Israel	1	1196	3	0.30 (0.10–0.80)	−	−	−
Japan	4	4033	80	2.00 (1.00–4.20)	27.61	89.14	*p* < 0.0001
Mongolia	3	727	35	5.10 (2.20–11.50)	11.87	83.15	*p* = 0.003
Thailand	1	12,184	1	0.00 (0.00–0.10)	−	−	−
Nigeria	1	700	0	0.00 (0.00–0.00)	−	−	−
United States	12	22,300	806	4.30 (3.00–6.20)	237.33	94.95	*p* < 0.0001
Canada	1	248	4	1.60 (0.60–4.20)	−	−	−

I^2^: inverse variance; *Q*-*p*: Cochran’s; CI: confidence interval; MIR: minimum infection rate. Measure of heterogeneity: the weighted sum of squared differences between individual study effects and the pooled effect across studies.

## Data Availability

The data presented in this study are available in the current article and the supplementary material.

## Supplementary Materials

The following are available online at https://www.mdpi.com/2076-0817/10/2/230/s1, Table S1: JBI Critical appraisal checklist for studies reporting prevalence data; Table S2: Quality assessment scores for eligible studies.
