# Supplementary material for: Global Distribution of Babesia Species in Questing Ticks: A Systematic Review and Meta-Analysis Based on Published Literature
Source: Pathogens. 2021 Feb 19;10(2):230. doi: 10.3390/pathogens10020230 (PMC7926846; doi:10.3390/pathogens10020230)
Supplement: Supplementary file 1 [file pathogens-10-00230-s001.zip › Table S2. Quality assessment scores for eligible studies.pdf]

Supplementary Table S2: Quality assessment scores for eligible studies

Study Name [Reference No.]	Q1	Q2	Q3	Q4	Q5	Q6	Q7	Q8	Q9	Overall score X/9 (% Score)
(Blaschitz et al. 2008)	1	1	1	0	0	1	1	0	NA	5 (55.6)
(Reye et al. 2013)	1	1	1	0	1	1	1	1	NA	7 (77.8)
(Klitgaard et al. 2019)	1	1	1	0	1	1	1	1	NA	7 (77.8)
(Sormunen et al. 2020)	1	1	1	0	1	1	1	1	NA	7 (77.8)
(Sormunen et al. 2018)	1	1	1	1	1	1	1	1	NA	8 (88.9)
(Bonnet et al. 2013)	1	1	1	0	1	1	1	1	NA	7 (66.7)
(Cotte et al. 2010)	1	1	1	1	1	1	1	1	NA	8 (66.7)
(Halos et al. 2005)	1	1	1	1	1	1	1	1	NA	8 (88.9)
(Jouglin et al. 2017)	1	1	1	1	1	1	1	1	NA	8 (77.8)
(Lejal et al. 2019)	1	1	1	1	1	1	1	1	NA	8 (88.9)
(Reis et al. 2011)	1	1	1	1	1	1	1	1	NA	8 (88.9)
(Eshoo et al. 2014)	1	1	1	0	1	1	1	1	NA	8 (88.9)
(Franke et al. 2010)	1	1	1	0	1	1	1	1	NA	8 (88.9)
(Franke et al. 2011)	1	1	1	0	1	1	1	1	NA	8 (88.9)
(Hartelt et al. 2004)	1	1	1	1	1	1	1	1	NA	8 (88.9)
(Hildebrandt et al. 2010)	1	1	1	1	1	1	1	1	NA	8 (88.9)
(Hildebrandt et al. 2011)	1	1	1	1	1	1	1	1	NA	8 (88.9)
(Overzier, Pfister, Thiel, et al. 2013)	1	1	1	1	1	1	1	1	NA	8 (88.9)
(Overzier, Pfister, Herb, et al. 2013)	1	1	1	1	1	1	1	1	NA	8 (88.9)
(Schorn et al. 2011)	1	1	1	1	1	1	1	1	NA	8 (88.9)
(Silaghi et al. 2012)	1	1	1	1	1	1	1	1	NA	8 (88.9)
(Silaghi, Weis, and Pfister 2020)	1	1	1	1	1	1	1	1	NA	8 (88.9)
(Kohn et al. 2019)	1	1	1	1	1	1	1	1	NA	8 (88.9)
(Sprong et al. 2019)	1	1	1	0	1	1	0	1	NA	6 (66.7)
(Egyed et al. 2012)	1	1	1	0	0	1	0	1	NA	5 (55.6)
(Hornok et al. 2016)	1	1	1	1	1	1	1	1	NA	8 (88.9)
(Capelli et al. 2012)	1	1	1	1	1	1	1	1	NA	8 (88.9)
(Cassini et al. 2010)	1	1	1	0	0	1	1	1	NA	6 (66.7)
(Floris et al. 2009)	1	1	1	1	0	1	1	1	NA	7 (77.8)
(Piccolin et al. 2006)	1	1	1	0	0	0	1	1	NA	5 (55.6)
(Capligina et al. 2016)	1	1	1	1	1	1	1	1	NA	8 (88.9)
(Radzijevskaja et al. 2018)	1	1	1	1	1	1	1	1	NA	8 (88.9)
(Oines et al. 2012)	1	1	1	1	1	1	1	1	NA	8 (88.9)
(Radzijevskaja, Paulauskas, and Rosef 2008)	1	1	1	1	1	1	1	1	NA	8 (88.9)
(Žygutienė et al. 2008)	1	1	1	1	0	1	1	1	NA	7 (77.8)
(Wielinga et al. 2009)	1	1	1	1	0	1	1	1	NA	7 (77.8)
(Asman et al. 2015)	1	1	1	0	0	1	1	1	NA	6 (66.7)
(Cieniuch, Stanczak, and Ruczaj 2009)	1	1	1	1	1	1	1	1	NA	8 (88.9)
(Karbowiak et al. 2014)	1	1	1	0	0	1	1	0	NA	5 (55.6)
(Pieniazek, Sawczuk, and Skotarczak 2006)	1	1	1	1	0	1	1	1	NA	7 (77.8)

(Sinski et al. 2006)	1	1	1	1	1	1	1	1	NA	8 (88.9)
(Skotarczak, Wodecka, and Cichocka 2002)	1	1	1	1	1	1	1	1	NA	8 (88.9)
(Stanczak et al. 2015)	1	1	1	1	1	1	1	1	NA	8 (88.9)
(Welc-Faleciak et al. 2012)	1	1	1	0	0	1	1	1	NA	6 (66.7)
(Wojcik-Fatla et al. 2012)	1	1	1	1	1	1	1	1	NA	8 (88.9)
(Wojcik-Fatla et al. 2015)	1	1	1	1	1	1	1	1	NA	8 (88.9)
(Zajac et al. 2017)	1	1	1	1	0	1	1	1	NA	7 (77.8)
(Wojcik-Fatla et al. 2009)	1	1	1	1	0	1	1	1	NA	7 (77.8)
(Stanczak et al. 2004)	1	1	1	1	0	1	1	1	NA	7 (77.8)
(Katargina et al. 2011)	1	1	1	0	0	1	1	1	NA	6 (66.7)
(Maia et al. 2014)	1	1	1	1	1	1	1	1	NA	8 (88.9)
(Santos-Silva et al. 2017)	1	1	1	1	1	1	1	1	NA	8 (88.9)
(Reye et al. 2010)	1	1	1	1	0	1	1	1	NA	7 (77.8)
(Ionita et al. 2013)	1	1	1	1	1	1	1	1	NA	8 (88.9)
(Karlsson and Andersson 2016)	1	1	1	1	0	1	1	1	NA	7 (77.8)
(Jaenson and Wilhelmsson 2019)	1	1	1	1	1	1	1	1	NA	8 (88.9)
(Alekseev, Semenov, and Dubinina 2003)	1	1	1	0	0	1	1	1	NA	6 (66.7)
(Movila et al. 2014)	1	1	1	0	0	1	1	1	NA	6 (66.7)
(Rar et al. 2005)	1	1	1	0	0	1	1	1	NA	6 (66.7)
(Rar et al. 2011)	1	1	1	0	0	1	1	1	NA	6 (66.7)
(Rar et al. 2017)	1	1	1	0	0	1	1	1	NA	6 (66.7)
(Duh et al. 2006)	1	1	1	1	1	1	1	1	NA	8 (88.9)
(Hamsikova et al. 2016)	1	1	1	1	1	1	1	1	NA	8 (88.9)
(Svehlova et al. 2014)	1	1	1	1	0	1	1	1	NA	7 (77.8)
(Duh, Petrovec, and Avsic-Zupanc 2001)	1	1	1	1	0	1	1	1	NA	7 (77.8)
(Rudolf et al. 2005)	1	1	1	1	1	1	1	1	NA	8 (88.9)
(Cochez et al. 2012)	1	1	1	0	0	1	1	1	NA	6 (66.7)
(Jongejan et al. 2015)	1	1	1	0	1	1	1	1	NA	7 (77.8)
(Garcia-Sanmartin et al. 2008)	1	1	1	1	1	1	1	1	NA	8 (88.9)
(Casati et al. 2006)	1	1	1	0	1	1	1	1	NA	7 (77.8)
(Gigandet et al. 2011)	1	1	1	1	1	1	1	1	NA	8 (88.9)
(Lommano et al. 2012)	1	1	1	1	0	1	1	1	NA	7 (77.8)
(Oechslin et al. 2017)	1	1	1	1	1	1	1	1	NA	8 (88.9)
(Schaarschmidt et al. 2013)	1	1	1	1	1	1	1	1	NA	8 (88.9)
(Didyk et al. 2017)	1	1	1	1	0	1	1	1	NA	7 (77.8)
(Brinkmann et al. 2019)	1	1	1	1	0	1	1	1	NA	7 (77.8)
(Orkun et al. 2020)	1	1	1	1	1	1	1	1	NA	8 (88.9)
(Omar Abdallah et al. 2016)	1	1	1	1	1	1	1	1	NA	8 (88.9)
(Li et al. 2020)	1	1	1	1	1	1	1	1	NA	8 (88.9)
(Niu et al. 2017)	1	1	1	1	0	1	1	1	NA	7 (77.8)
(Harrus et al. 2011)	1	1	1	0	0	1	1	1	NA	6 (66.7)
(Masatani et al. 2017)	1	1	1	1	1	1	1	1	NA	8 (88.9)
(Zamoto-Niikura et al. 2012)	1	1	1	1	1	1	1	1	NA	8 (88.9)
(Sivakumar et al. 2014)	1	1	1	1	1	1	1	1	NA	8 (88.9)

(Zamoto-Niikura et al. 2016)	1	1	1	1	1	1	1	1	NA	8 (88.9)
(Battsetseg et al. 2001)	1	1	1	0	0	1	1	1	NA	6 (66.7)
(Karnath et al. 2016)	1	1	1	1	1	1	1	1	NA	8 (88.9)
(Tuvshintulga et al. 2015)	1	1	1	1	0	1	1	1	NA	7 (77.8)
(Wattanamethanont, Kaewthamasorn, and Tiawsirisup 2018)	1	1	1	1	1	1	1	1	NA	8 (88.9)
(Reye et al. 2012)	1	1	1	1	1	1	1	1	NA	8 (88.9)
(Adelson et al. 2004)	1	1	1	0	0	1	1	1	NA	6 (66.7)
(Edwards et al. 2015)	1	1	1	0	0	1	1	1	NA	6 (66.7)
(Piesman et al. 1986)	1	1	1	1	1	1	1	1	NA	8 (88.9)
(Varde, Beckley, and Schwartz 1998)	1	1	1	0	0	1	1	1	NA	6 (66.7)
(Steiner et al. 2008)	1	1	1	1	0	1	1	1	NA	7 (77.8)
(Steiner et al. 2006)	1	1	1	0	0	1	1	1	NA	6 (66.7)
(Edwards et al. 2019)	1	1	1	1	1	1	1	1	NA	8 (88.9)
(Venclíková et al., 2016)	1	1	1	1	1	1	1	1	NA	8 (88.9)
(Russart, Dougherty, and Vaughan 2014)	1	1	1	1	1	1	1	1	NA	8 (88.9)
(Hutchinson et al. 2015)	1	1	1	1	1	1	1	1	NA	8 (88.9)
(Prusinski et al. 2014)	1	1	1	1	1	1	1	1	NA	8 (88.9)
(Aliota et al. 2014)	1	1	1	0	1	1	1	1	NA	7 (77.8)
(Hersh et al. 2014)	1	1	1	1	1	1	1	1	NA	8 (88.9)
(Milnes et al. 2019)	1	1	1	1	1	1	1	1	NA	8 (88.9)
